# Supplementary material for: Regulation of miR319b-Targeted SlTCP10 during the Tomato Response to Low-Potassium Stress
Source: Int J Mol Sci. 2023 Apr 11;24(8):7058. doi: 10.3390/ijms24087058 (PMC10138608; doi:10.3390/ijms24087058)

Supporting Information

Table S1 PCR primer sequences used for vector construction and qRT-PCR analysis primer sequences.

Name	Sequence(5'-3')
GeneRacer RNA Oligo	CGACUGGAGCACGAGGACACUGACAUGGACUGAAGGAGUAGAAA
**GeneRacer 5' Primer**	CGACTGGAGCACGAGGACACTGACA
GeneRacer 5'Nested primer	GGACACTGACATGGACTGAAGGAGTA
**SlTCP10-RACE**	ATTAGGAGAAGCAGAGTCATGATGT
**SlTCP10-RACE_nested**	TGGCACTTGAAAATCAGTAGTAAAA
**SlTCP1-RACE**	GTCAAGAAGCAGTAAAAAATGTAGC
**SlTCP1-RACE_nested**	AAATGTAGCCTGCTCCTCTTCCCCT
**TCP10-F (vector construction)**	gccatatg ATGAAGAGCGCAACTGGAGGAG
**TCP10-R (vector construction)**	gcctcgag GCATTAGGAGAAGCAGAGTCA
**SlJA2-F(vector construction)**	cagtTTTCGCAGCATCTAACGAGCTCTTCGgatcatgggtgttcaagaaaaaga

**SlJA2-R(vector construction)**	cgatTTTCGCAGCATCTAACGAGCTCTTCGctactgcttgaacccgagat
**JA2 promoter-F**	TCTATCCGTCCCTATTTAGTT
**JA2 promoter-R**	TAATAGCCGACCTTATCG
**Bar-F**	GAAGTCCAGCTGCCAGAAA
**Bar-R**	CACCATCGTCAACCACTACAT
**SlJA2-CR-gRNA1**	TGCAATTAAGTTTACCACC
**SlJA2-CR-gRNA2**	CTAGACCGAATCGAGTAGC
**miR319b-RT**	GTCGTATCCAGTGCAGGGTCCGAGGTATTCGCACTGGATACGACAGGGAG
**miR319b-F**	GAGTGTTGACTGAAGGGACA
**miR319b-R**	GCAGGGTCCGAGGTATTC
**JA2-F**	CAAAGCTAGATGAATGGGTGCT
**JA2-R**	GAATTTCTCGGCCTTGAGAGA
**JA2p-EMSA**	TTTATTTAGATTCATGTATTTGGTCCAAAACTTTACTGCTCGATAAGGTC

**Actin**	ACCGAAGCCCCTCTTAACCC
**(reference)**	GTATGGCTGACACCATCACC
**U6**	CATCCGATAA AATTGGAACGA
**(reference)**	TTTGTGCGTGTCATCCTTGCG

Figure S1 The plant phenotype of JZ34 and OE-SlmiR319 with low potassium treatment 21days.

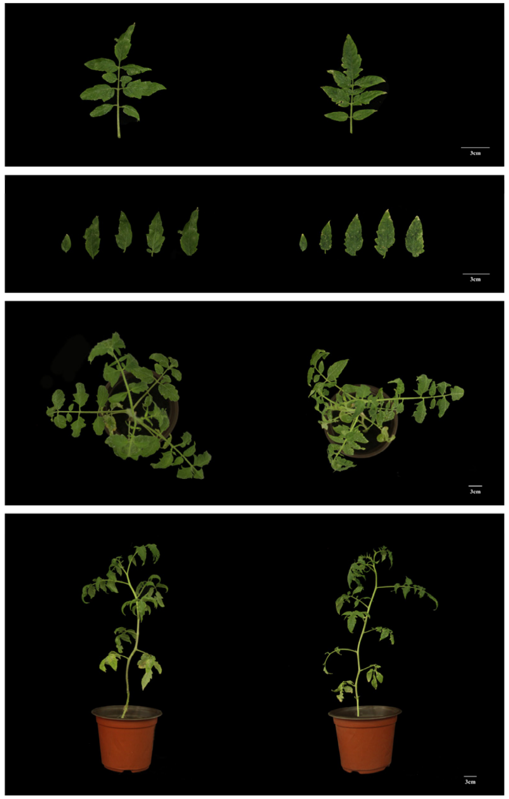

JZ34 **OE-*SlmiR319b***

Figure S2 The root hair phenotype of sit mutants and WT under the normal condition and low potassium (LK) treatment 7d.

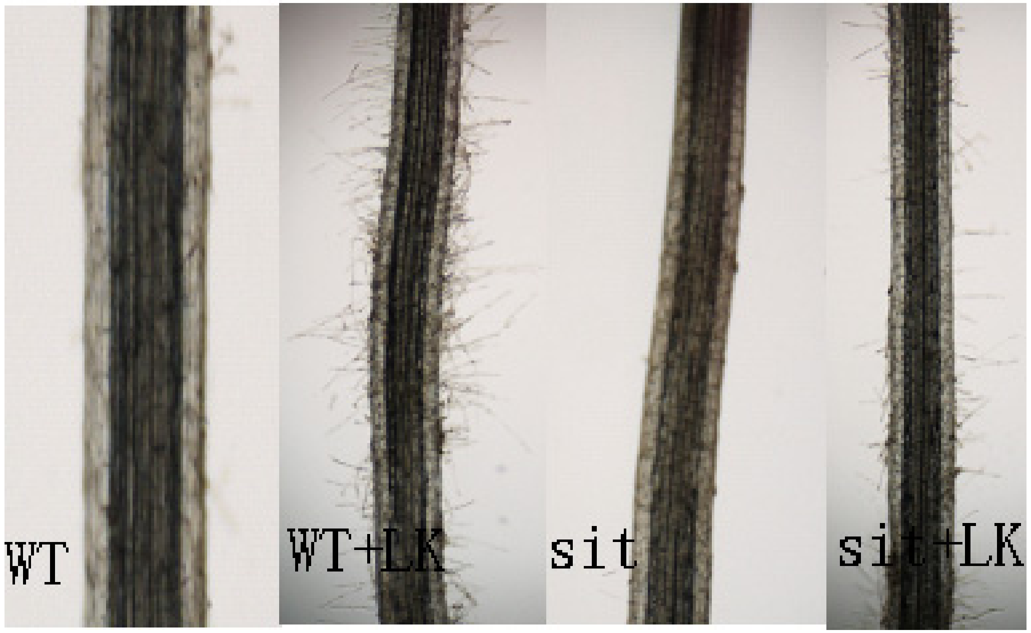

Supplement: Supplementary file 1 [file ijms-24-07058-s001.zip › ijms-2276411-supplementary.pdf]
